# Acute cognitive effects of single-dose intravenous ketamine in major depressive and posttraumatic stress disorder

**DOI:** 10.1038/s41398-021-01327-5

**Published:** 2021-04-08

**Authors:** Margaret T. Davis, Nicole DellaGiogia, Paul Maruff, Robert H. Pietrzak, Irina Esterlis

**Affiliations:** 1 Yale University School of Medicine, Department of Psychiatry, New Haven, USA; 2 Yale University, Department of Psychology, New Haven, USA; 3 U.S. Department of Veterans Affairs, National Center for Posttraumatic Stress Disorder, Washington, DC, USA; 4 Cogstate, Ltd., Fitzroy, Australia

**Keywords:** Human behaviour, Molecular neuroscience

## Abstract

Intravenous (IV) subanesthetic doses of ketamine have been shown to reduce psychiatric distress in both major depressive (MDD) and posttraumatic stress disorder (PTSD). However, the effect of ketamine on cognitive function in these disorders is not well understood. To address this gap, we examined the effect of a single dose of IV ketamine on cognition in individuals with MDD and/or PTSD relative to healthy controls (HC). Psychiatric (*n* = 29; 15 PTSD, 14 MDD) and sex- age- and IQ matched HC (*n* = 29) groups were recruited from the community. A single subanesthetic dose of IV ketamine was administered. Mood and cognitive measures were collected prior to, 2 h and 1 day post-ketamine administration. MDD/PTSD individuals evidenced a large-magnitude improvement in severity of depressive symptoms at both 2-hours and 1 day post-ketamine administration (*p*’s < .001, Cohen *d*’s = 0.80–1.02). Controlling for baseline performance and years of education, IV ketamine induced declines in attention (ATTN), executive function (EF), and verbal memory (VM) 2 h post-administration, all of which had resolved by 1 day post-ketamine across groups. The magnitude of cognitive decline was significantly larger in MDD/PTSD relative to HC on attention only (*p* = .012, *d* = 0.56). Ketamine did not affect working memory (WM) performance. Cognitive function (baseline, change from baseline to post-ketamine) was not associated with antidepressant response to ketamine. Results suggest that while ketamine may have an acute deleterious effect on some cognitive domains in both MDD/PTSD and HC individuals, most notably attention, this reduction is transient and there is no evidence of ketamine-related cognitive dysfunction at 1 day post-administration.

## Introduction

Ketamine is a *N*-methyl-d-aspartate (NMDA) receptor antagonist that acts as a dissociative anesthetic^1^. Intravenous (IV) administration of ketamine rapidly reduces symptoms of both major depressive disorder (MDD)^2–4^ and posttraumatic stress disorder (PTSD)^5,6^ including suicidal ideation^7–9^. The ability to provide fast and robust symptom relief has led to substantial off-label use of IV ketamine^4^ in individuals with MDD, who frequently experience refractory symptoms despite treatment with FDA approved antidepressant medication^10^. It has also lead to widespread off-label use in PTSD where effective pharmacotherapies are limited^11^. Consistent with these therapeutic benefits, an intranasal formulation of a ketamine enantiomer, esketamine, was approved in 2019 as a breakthrough therapy for treatment-resistant depression (TRD)^12,13^. However, given the widespread and growing use of IV ketamine as a therapeutic agent, it is important to understand the risks associated with the drug^14,15^. Cognitive dysfunction is one important central nervous system (CNS)-related adverse outcome associated with both acute and chronic IV ketamine use^16,17^. Such risk is especially concerning for therapeutic use of IV ketamine in MDD and PTSD where ketamine-induced cognitive dysfunction could add to the subtle (i.e., *d* ~ 0.5) impairments in attention, memory, and concentration that characterize both disorders^18,19^. Thus, a thorough understanding of the effect of IV ketamine administration on cognition is essential to clinicians considering it as an intervention option.

In experimental animal^20^ and healthy human^21–23^ studies, systematic IV administration of high-dose ketamine is an established model for transient psychosis within which the negative cognitive effects are always observed, and in many studies have been the central focus. High-dose ketamine has also demonstrated neurotoxic effects in both mature^24,25^ and developing animals^26,27^, and chronic abuse of ketamine is associated with decline across many domains of cognition^28^. In human experimental models, IV ketamine-related cognitive decline has been shown to resolve with clearance of the drug^29,30^. Similarly, the impaired cognition observed in chronic ketamine abusers can resolve with abstinence^31^. Unfortunately, the extent to which these data can guide decisions about CNS-associated risks associated with IV ketamine treatment in MDD or PTSD is limited, as few studies examining the relationship between ketamine and cognition have included MDD groups^17,32^, none have included a PTSD group, and the IV ketamine doses studied have generally been higher than those shown to be beneficial to depressive symptoms (e.g., subanesthetic doses; recommended dose of 0.5 mg/kg body weight^33^). An examination of the effects on cognition of therapeutic doses of IV ketamine in MDD and PTSD are needed to inform models of CNS risk associated with the drug.

Although several studies have conducted examination of ketamine-induced cognitive changes in individuals with MDD, results of such studies have been inconsistent with some reporting no effect on cognition^34–36^, some reporting a decline in episodic memory and recall^29,37–39^, semantic processing^38^ and sustained attention^29,40,41^, and others an improvement in cognition^42–45^ including in verbal and visual memory^43^, processing speed, and working memory (WM)^44^. The most frequently replicated and robust findings in MDD concern deficits in verbal memory (VM), attention, and executive functioning^46–49^. To date, no known study has examined the effect of IV ketamine on cognition in PTSD. As these studies utilized therapeutic doses of IV ketamine in individuals with similar characteristics, observed inconsistencies likely reflect differences in methodology such as (i) the time between ketamine administration and cognitive testing which has varied between 40 min^50^ and 26 days^45^, (ii) the use of single^35,43,50^ vs. multiple doses^34,44,45^, (iii) whether ketamine effects were determined with reference to a placebo or active control^43,50^, compared to a saline control^37,41^ or even no control^34^, or (iv) the extent to which the outcome measures used had demonstrated sensitivity^51,52^ to ketamine-related cognitive decline^43,45,50^. Therefore, to provide a foundation for understanding CNS risk for IV ketamine at therapeutic doses in MDD and PTSD, it is important to understand (a) the nature and magnitude of acute effects of the drug on cognition and (b) how these compare to those which occur in matched healthy adults assessed over the same time period. Furthermore, these effects should be determined using tests that have demonstrated sensitivity to the acute and chronic cognitive effects of IV ketamine.

Toward this end, we conducted an experiment to investigate the acute effects of therapeutic (subanesthetic) doses of IV ketamine on cognitive function in adults with MDD and PTSD. Prior to, and then at 2h and 1 day following ketamine administration attention (ATTN), working memory (WM), executive function (EF), and verbal memory (VM) were assessed using tests with demonstrated sensitivity to both the acute and chronic effects of ketamine^44,53^. Given the questionable validity of placebo conditions for IV ketamine^54^, we made inferences about the acute effect of the drug in the psychiatric group by comparing ketamine-related cognitive changes to performance in the clinical group to changes that occurred for the same doses in healthy matched controls. First, we hypothesized that IV ketamine administration would be associated with acute improvement in mood symptoms in individuals with MDD and PTSD. Second, we hypothesized that IV ketamine would be associated with a decline in cognition relative to baseline at 2 h post-administration but would return to baseline levels 1 day post-administration. Finally, we explored whether the level of symptoms at baseline within the psychiatric group influences the magnitude of any cognitive responses observed.

## Materials and Methods

### Participants

General inclusion criteria for the study were as follows: (1) age 18–60 years old; (2) English speaking (to facilitate consent); (3) no recent regular history of psychiatric medication use. For the psychiatric group, participants were (1) individuals who met *DSM-IV-TR*^55^ diagnostic criteria for MDD (*n* = 14) and/or PTSD (*n* = 15) based on the Standardized Clinical Interview for DSM-IV (SCID)^56^, (2) were currently in a major depressive episode (for those with a diagnosis of MDD), (3) had no other psychiatric conditions (with the exception of anxiety disorders), and (4) were free of psychotropic medications for at least 2 months at the time of ketamine injection. For the healthy control (HC) group, participants (*n* = 29) met general inclusion criteria listed above, met criteria for no psychiatric diagnoses either currently or historically, and had no first degree relatives with psychiatric diagnoses. Five participants with MDD met criteria for one or more anxiety disorder on the SCID (e.g., OCD, panic disorder), and ten participants with MDD reported having experienced a traumatic event meeting DSM criterion A1 for PTSD. Eight participants with PTSD also met criteria for comorbid MDD. Groups were matched for age, sex, and smoking status (see Table 1 for detailed demographic information). This study was approved by the Yale University Institutional Review Board and written informed consent was obtained from all participants prior to data collection.

**Table 1:** Demographic and clinical characteristics of the HC and MDD/PTSD group.

	Healthy controls	MDD/ PTSD	Test of difference	*p*
*N*	29	29		
	Mean (SD) or *n* (%)	Mean (SD) or *n* (%)		
Age	33.3 (12.1)	36.1 (13.8)	1.03	0.31
Female sex	16 (55.0)	18 (62.1)	0.01	0.98
Body mass index	27.8 (8.3)	27.0 (5.3)	0.01	0.98
Race (White)	17 (58.6)	17 (58.6)	0.01	0.98
Years of education**	15.4 (1.9)	14.0 (1.7)	8.78	0.004
Standardized IQ score	108.3 (15.6)	101.5 (22.0)	0.35	0.55
MADRS (baseline)**	0.5 (1.2)	22.6 (9.2)	154.00	<0.001
BDI-II (baseline)**	0.9 (1.9)	22.6 (12.5)	137.86	<0.001
WM (pre-ketamine)	97.6 (9.6)	96.3 (8.6)	0.31	0.58
ATTN (pre-ketamine)	90.6 (10.8)	85.75 (12.3)	2.32	0.09
EF (pre-ketamine)	99.7 (12.8)	95.1 (14.4)	1.10	0.31
VM (pre-ketamine)	98.9 (10.5)	101.29 (10.0)	1.52	0.22
WM (2 h post)	96.7 (10.3)	97.0 (13.9)	0.01	0.91
ATTN (2 h post)*	90.0 (11.2)	79.6 (13.6)	9.32	0.004
EF (2 h post)*	96.1 (11.0)	89.2 (13.3)	4.24	0.04
VM (2 h post)	94.8 (12.5)	91.2 (13.1)	1.07	0.31
WM (1 day post)	97.1 (11.8)	99.6 (20.8)	0.27	0.61
ATTN (1 day post)	91.9 (12.4)	86.06 (13.3)	2.55	0.12
EF (1 day post)	100.89 (9.26)	94.9 (19.8)	1.10	0.31
VM (1 day post)	102.6 (7.18)	97.6 (10.4)	3.89	0.06

Underline denotes groups with significant differences based on post-hoc testing.

*MADRS* Montgomery–Asberg Depression Rating Scale, *BDI-II* Beck depression inventory II, *ATTN* attention/ psychomotor speed composite score, *WM* learning/ working memory composite score.

Significant group difference: *p  < 0.01; **p < 0.001.

### Ketamine administration

Ketamine IV was administered by a licensed nurse and attending physician. All IV ketamine injections were completed in the context of a neuroimaging study and were completed at the Yale PET Center^57,58^. Participants were closely monitored both prior to and during the 3 h following ketamine injection. Vital signs (pulse, blood pressure, and oxygen saturation) were obtained before and after ketamine administration and during the ketamine infusion (at 5- to 10-min intervals). Racemic ketamine was obtained from the Yale–New Haven Hospital Pharmacy and administered to half the subjects in each group intravenously with an initial bolus of 0.23 mg/kg over 1 min followed by constant infusion of 0.58 mg/kg per hour over 1 h^59,60^. The remaining half of the subjects in each group received constant IV infusion of 0.5 mg/kg ketamine over 40 min. Of note, the slight alteration in dosing regimen were necessitated by subjects participation in two separate neuroimaging protocols (positron emission tomography studies with identical procedures for clinical and cognitive assessment, but utilizing different radiotracers). Participants did not significantly differ in any way (demographics, clinical variables, cognition) as a function of these minor alterations in dosing regimen.

### Mood assessments

The Structured Clinical Interview for DSM (IV)^61^ was administered at screening. The Hamilton Depression Rating Scale^62^, Beck Depression Inventory, 2nd edition^63^ (BDI-II), and the Montgomery–Åsberg Depression Rating Scale^64^ (MADRS) were used to assess participants’ mood at screening, before ketamine, 2 h post-ketamine administration, and 1 day post-ketamine administration). For the purpose of analyses, ketamine treatment responder status was defined using the previously validated minimum clinically important difference (MCID) on the BDI-II. That is, the value established in previous research as representing a clinically meaningful change in depressive symptoms following treatment (three points on the BDI-II^65,66^). Of note, MCID on the BDI-II in lieu of the MADRS was used in analyses in light of greater observed variability on the measure (only three individuals were classified as treatment nonresponders using the MADRS-MCID). Finally, the Weschler Test of Adult Reading^67^ was administered to assess premorbid intellectual functioning (IQ). Basic demographic information was also collected.

### Cognitive assessments

Cognitive function was measured using tests from the Cogstate battery selected to measure domains of cognition relevant to both MDD and PTSD^68–70^. These tests included the Detection (DET) test of psychomotor function, which requires individuals to respond to the presentation of a visual stimulus where the speed of the response was the performance measure; the Identification (IDN) test of visual attention, which requires individuals to decide the color of a visual stimulus where the speed of the response was the performance measure; the One Back (ONB) test of WM, which requires individuals to determine whether the stimulus presented was identical to the one that had been presented on the immediately previous trial and where the speed of the response was the performance measure; the One Card Learning (OCL) test of visual learning, which requires individuals to decide if a visual stimulus presented had been presented previously in the test and where the accuracy of responses was the performance measure; the Groton Maze Learning Test (GMLT) of EF, which requires individuals to find and learn the location of a 28-step pathway hidden under a 10 × 10 matrix of tiles over five consecutive trials, where the main performance measure was the number of errors made; and the International Shopping List test (ISLT) of learning and memory, which requires individuals to learn a list of 12 shopping list items over three consecutive trials (acquisition) as well as recall those items after a delay (memory) where the number of words recalled in each aspect were the main performance measures. These tests have been described in detail previously in the context of their use in experimental medicine contexts^44,71–73^. The tests used here were selected specifically for their ability to be readministered at short retest intervals without practice effects^72^, and demonstrated sensitivity to, and the long-term cognitive effects of subanesthetic IV ketamine^44^. Test were administered according to standard instructions with the presentation of stimuli and recording of responses controlled by a laptop computer under supervision of a trained rater. The complete assessment required less than 30 min.

### Data processing

Prior to analyses, the main performance measure for each cognitive outcome measure was standardized using the mean and standard deviation of age stratified (18–34, 35–49, 50–59, and 60–69 years old) normative data^74,75^. Because the numerical direction for abnormal scores was different for tests that used accuracy (i.e., higher values indicate less impairment) and speed (i.e., higher values indicate greater impairment), the signs of standardized scores were adjusted so that negative signs indicated performance worse than the relevant age matched mean and positive scores indicated performance better than the age matched mean. To reduce the number of outcome measures and also the potential for Type I error, the main outcome measures from the seven cognitive tests were compiled into two composite scores^73^ and two individual scores. An attention composite was computed by averaging the age-standardized scores from the DET and IDN test. A WM composite was computed by averaging the age-standardized scores on the OCL and ONB tests. For each subject, the attention composite and the WM composite was re-standardized with reference to the mean and SD of the same composite scores derived from the normative data. The four age-standardized outcome measures (hereafter termed cognitive outcomes) were then organized into the main cognitive domain they measured with attention (ATTN) measured by the DET/IDN composite, WM measured by the OCL/ONB composite, EF measured by the GMLT, and VM measured by the ISLT and submitted to statistical analyses.

### Data analysis

Data analyses proceeded in four steps. First, chi-squared tests and analyses of variance (ANOVAs) were conducted to compare MDD/PTSD and HC groups with respect to demographic characteristics (age, sex, years of education, IQ—to verify matching). Of note, the decision to combine MDD and PTSD groups was made for two reasons: (1) based on review of available prior literature we expected to observe a similar pattern of results in MDD and PTSD; (2) in consideration of statistical power given modest sample sizes. As detailed below, we explored potential differences in MDD vs. PTSD in exploratory analyses. Second, to evaluate the effect of ketamine on mood, a repeated-measures ANOVA was conducted with BDI-II score at each timepoint, diagnostic group, and the cognition*group interaction entered into the model. Third, cognition in both groups at 2 h (hypothesis 1) and 1 day (hypothesis 2) post-administration, five analysis of covariance (ANCOVA) models were conducted (one for each cognitive outcome, one for mood). Change scores (baseline vs. each timepoint) were computed and entered dependent variables, and baseline mood or cognitive score/composite and level of education were entered as covariates. Third, we conducted exploratory analyses to examine the relationship between baseline cognition and ketamine responder status. Specifically, multivariate analysis of covariance (MANCOVA) was used to compare cognitive test performance in HC, MDD, and PTSD groups and across ketamine responders and nonresponders. Group (HC, MDD, PTSD) and ketamine response (defined as meeting the MCID on the BDI-II following ketamine administration at each timepoint) were entered as a fixed factor, and their interaction was entered into the model. Cohen’s *d* was computed to estimate effect sizes of group differences in cognitive test scores. Statistical significance was determined using the Benjamini–Hochberg procedure^76^.

## Results

### Sample characteristics

**Fig. 1:**
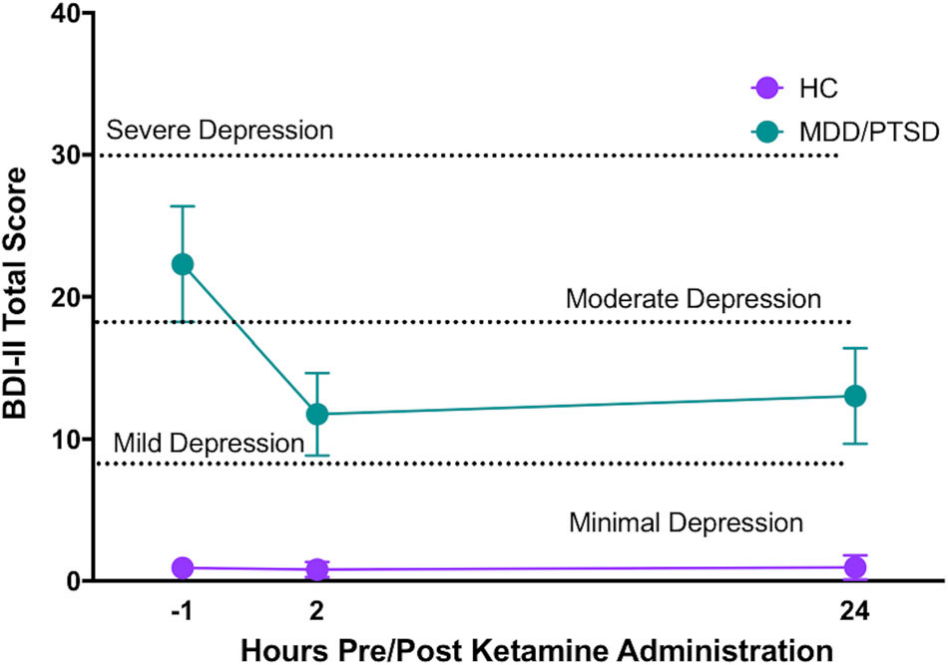
Mean depression symptom severity scores (self-reported on the Beck Depression Inventory II) across timepoints in healthy control participants (HC) and participants with major depressive disorder (MDD) and posttraumatic stress disorder (PTSD). Error bars indicate 95% confidence intervals. Dotted lines separate categories of depression symptom severity. BDI-II Beck Depression Inventory II.

Table 1 shows demographic and clinical characteristics of the sample. The MDD/PTSD and HC groups did not differ significantly with respect to age, sex, IQ, or BMI. There was a significant difference in years of education between groups (*t* = 8.78, *p* = 0.004). Mean depressive symptom ratings prior to ketamine administration were indicative of moderate depression in the MDD/PTSD group based on both self-report and clinician-administered measures (MADRS *M* = 22.7 ± 4.7; BDI-II *M* = 22.0 ± 8.7, see Fig. 1). As expected, depressive symptoms were significantly greater in MDD/PTSD group than in the HC group at all timepoints (*p* < 0.001). None of the cognition scores or change scores qualified as outliers using a standard of 2.5 times the standard deviation of each variable for identification and classification. As such, no outliers were fenced or removed from the dataset prior to analysis.

### Effect of ketamine on mood symptoms

Repeated-measures ANOVA showed a significant interaction of MDD/PTSD status and depressive symptom severity across timepoints (*F* = 11.39, *p* < .001, *d* = 0.86; See Fig. 1); mean depressive symptom severity decreased from moderate depression at baseline to mild depression 2 h and 1 day post-ketamine administration in the MDD/PTSD group.

Exploratory analyses were also performed to examine potential differences within the MDD/PTSD group on mood and demographic variables. Individuals with PTSD and MDD did not differ significantly on the BDI-II at any timepoint, though both groups differed significantly from HC at all timepoints. On the clinician-administered MADRS, however, at 2 h post- ketamine administration individuals with PTSD reported significantly lower MADRS total scores than those with MDD (*M* difference = 7.1 points, *p* < .001, *d* = 0.99), and no longer differed significantly from HC. Otherwise, MADRS results matched those observed on the BDI-II. As discussed above, treatment responder/nonresponder status was defined based using MCID on the BDI-II computed based on data collected at the end of the ketamine day, and 1 day post- ketamine. MDD participants classified as responders to ketamine (*n* = 9, 64.3%) improved by an average of 11.6 points (52.5%) 2 h post-ketamine, and 8 points (36.4%) 1 day post. Results among PTSD responders (*n* = 11, 73.3%) were equivalent; an average improvement of 11.8 points (53.3%) 2 h post- ketamine and 9.3 points (42.2%) 1 day post.

### Cognition pre- and post-ketamine

**Fig. 2:**
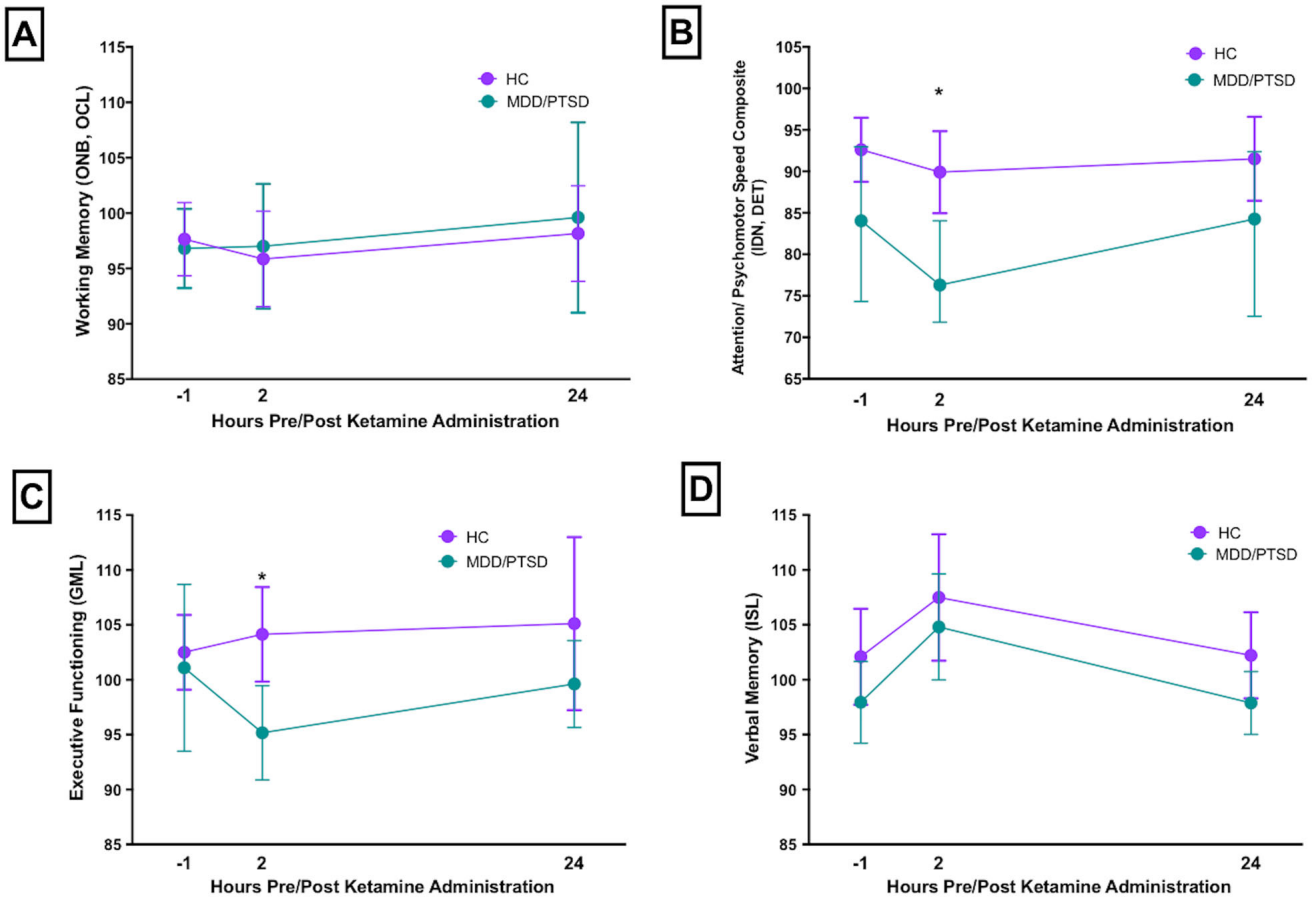
Mean and 95% confidence intervals for normalized Cogstate scores/composite scores across the three study timepoints: pre-ketamine (prior to ketamine administration); 2 h post-ketamine; 24 h post-ketamine administration across groups. HC healthy control, MDD major depressive disorder, PTSD posttraumatic stress disorder, IDN identification task, DET detection task, OCL one card learning task, ONB, one-back task, GML Groton maze learning test, ISL International Shopping List. *p < .01.

**Fig. 3:**
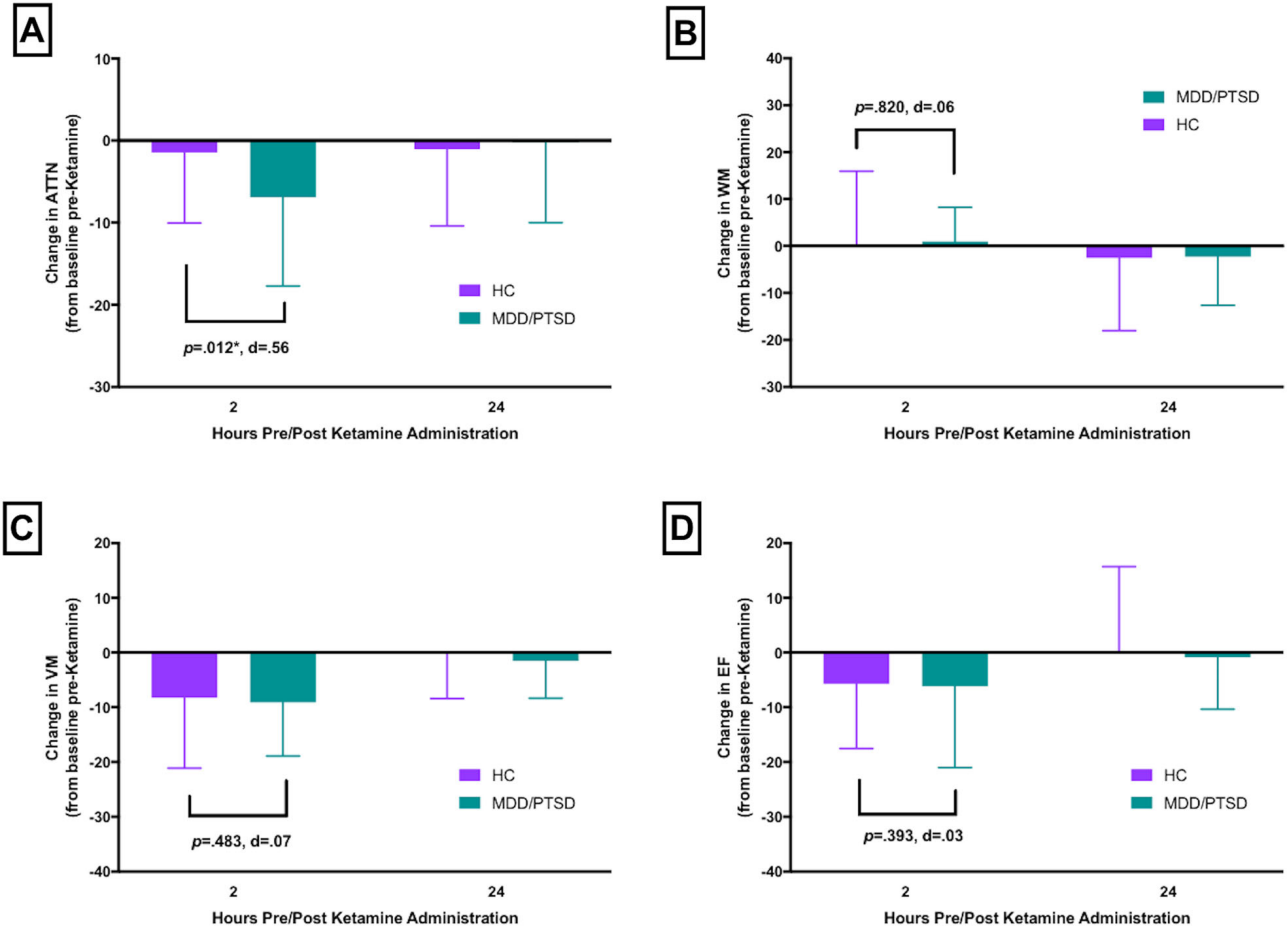
Mean and SD for change scores at each timepoint relative to baseline for each Cogstate score/composite score. HC healthy control, MDD major depressive disorder, PTSD posttraumatic stress disorder, ATTN attention composite score, WM working memory composite score, EF executive functioning, VM verbal memory. *p < .01.

Group means for each cognitive outcome measure in the HC and MDD/PTSD groups at baseline, 2 h and 1 day post-ketamine are shown in Fig. 2A–D. Results from ANCOVA analyses examining ketamine-induced changes in cognition at each timepoint controlling for baseline cognitive test performance are shown in Fig. 3. In HC, administration of IV ketamine induced a decline in performance on ATTN, VM, and EF at 2 h post-administration. The observed decline resolved 1 day post-ketamine, as evidenced by return to baseline functioning (See Fig. 3A, C, D). In the MDD/PTSD group, administration of IV ketamine induced a decline in the same domains (ATTN, VM, and EF). The magnitude of the decline was greater in MDD/PTSD relative to HC in ATTN only (*F* = 6.99, *p* = .012, *d* = 0.56; Fig. 3A). As in HC, the observed decline resolved 1 day post-ketamine. In contrast, ketamine administration did not affect performance on WM in either group (Fig. 3B).

### Exploratory analyses: Cognition and treatment response

Exploratory analyses were conducted to (1) examine potential differences between individuals with MDD and PTSD on cognitive variables and (2) facilitate examination of the relationship between baseline cognitive functioning and response to ketamine. For these analyses, the clinical group was divided to permit examination of potential differences in ketamine responsiveness in PTSD vs. MDD. No significant differences were observed between individuals with PTSD and MDD on ATTN or VM. However, individuals with MDD performed significantly worse than those with PTSD on EF at 2 h post-ketamine administration, and on WM (*F* = 4.56, *p* = .031) 1 day post-ketamine.

No significant relationships were observed between baseline cognitive functioning, diagnostic status, and treatment responder status post-ketamine administration. Further analyses were conducted in order to investigate the relationship between change in cognitive functioning (from baseline to each post-ketamine timepoint) and response to ketamine, with similar results. No significant relationships were observed between change in cognitive functioning following treatment, diagnostic status, and treatment responder status post-ketamine administration.

## Discussion

Consistent with both study hypotheses and prior findings, significant improvement in depressive symptom severity was observed in individuals with both MDD and PTSD at both 2 h and 1 day post-ketamine administration. Our hypotheses regarding the effect of ketamine on cognition, however, were only partially supported. In the MDD/PTSD group, ATTN, EF, and VM performance declined 2 h post-ketamine administration, and then returned to baseline 1 day after dosing. In healthy controls this same pattern of change was observed for EF and VM, but not ATTN, which showed no significant decline. IV ketamine administration had no acute effects on working memory in either the MDD/PTSD or healthy adults.

Comparison of the acute ketamine related change between the MDD/PTD group and healthy adults indicated the effects on EF and VM were equivalent in magnitude, whereas ketamine related decline in ATTN was significant greater in the MDD/PTSD group. From a neuropsychopharmacological perspective the current results show that IV ketamine, given at established therapeutic doses, induces an acute and substantial decline in higher cognitive functions such as EF and VM in both MDD/PTSD and in healthy adults. In MDD/PTSD, this effect extends to attentional functions. In healthy adults, IV ketamine was not associated with any acute decline in attention or working memory. Importantly, all negative effects on cognition resolved completely following clearance of ketamine (i.e., 1 day after dosing).

The pattern of ketamine-related cognitive decline here is consistent with findings from previous studies which have examined the effects of the drug in MDD. This finding suggests that ketamine administration induces an acute reduction that was clinically meaningful and domain specific in MDD/PTSD. Examination of previous literature helps to put this finding in context; NMDA antagonists, including ketamine, have been observed to disrupt motor coordination in both human and preclinical research (e.g., induce symptoms including ataxia, catalepsy)^77,78^. Indeed, higher doses of ketamine, sufficient to induce an analgesic response, have been shown to induce acute deficits in attention and psychomotor functioning (reflected in steering variation and swerving on a driving simulator), which has been found to be sufficient to impair driving safety^79,80^. Thus, both the observed impairment in attention and psychomotor speed, and restriction of this reduction to the acute period following ketamine administration, is consistent with previous research.

Overall, these findings add to the growing literature on the relationship between subanesthetic ketamine and cognitive functioning, both acutely following administration and after allowing for clearance of the drug. Of further interest, we address this issue in a mixed sample of participants with MDD and PTSD for the first time. Interestingly, exploratory analyses suggested generally equivalent effects of ketamine on cognition (acute decline) in those with MDD and PTSD. With two exceptions (EF scores 2 h post-ketamine and VM scores 1 day post), results among individuals with MDD and PTSD were comparable. Cognition in those with MDD and PTSD on other measures did not differ significantly at any timepoint, or on EF and VM at any other timepoints. Further, a majority of participants in both diagnostic groups qualified as responders to ketamine based on both self-report and clinician-administered measures, supporting the utility of ketamine as a potential antidepressant treatment for both conditions. Nonetheless, understanding the relationship between ketamine and cognition remains essential to its safe use as a therapeutic agent in both MDD and PTSD. Some research has suggested that cognitive deficits may mediate the relationship between depression and functional impairment^81^, and that lasting cognitive impairment can impede functional recovery even following remission of MDD^82^. With the exception of one acute impairment specific to the domain of attention, we found that one IV administration of subanesthetic doses of ketamine—consistent with current clinical practice—did not substantively affect cognitive functioning. These findings contribute to a growing body of literature supporting the use of ketamine as a relatively safe and effective therapeutic agent for potentially treatment-resistant conditions including MDD and PTSD.

A number of methodological limitations warrant consideration in evaluating these results. First, the primary limitation of this study is the lack of randomized/controlled design and absence of a placebo condition. It is possible that other variables, including practice effects, fatigue, and stress (known to have a deleterious effect on cognitive functioning^83^) affected cognition during the course of the study. In the absence of a placebo condition, the potential impact of such variables cannot be quantified. Thus, the lack of a randomized, controlled design in this study limits conclusions. Second, the sample size was relatively small, limiting the variability and therefore possibly the representativeness of the sample. Notably, modest sample sizes are common among published evaluations of the relationship between ketamine and cognition to date, a fact which may contribute to variability in observed results (due to limited generalizability, possible confounds related to statistical power where not carefully managed). Third, the relationship between ketamine and cognitive functioning was only evaluated short-term over two timepoints. As such, conclusions concerning the likely effect of ketamine on cognition in the long term cannot be drawn. Likewise, conclusions concerning the immediate effects of ketamine on cognition and how rapidly they resolve cannot be drawn. Fourth, the cognitive battery used in our study was extensive, but not comprehensive. Observed variability in results between this and other comparable studies may be attributable in part to use of measures evaluating slightly different cognitive domains. Fifth, as noted one half of participants in each group received a slightly IV ketamine according to a slightly different dosing regimen (0.08 mg difference, 1 h vs. 40 min period), resulting in some procedural variability. Of note, careful examination of data prior to completion of primary analyses revealed no effects of dosing on clinical of cognitive variables. Similarly, no group differences in clinical or demographic variables were observed prior to ketamine administration. Thus, we are confident that procedural variability did not confound results. Further, conclusions are limited to a single acute IV administration of ketamine, and therefore provide limited insight concerning the effect of chronic administration on cognitive functioning. Finally, the population recruited was not currently taking psychiatric medication, and was thus not wholly representative of the psychiatric population in general.

As researchers and clinicians continue to explore, and potentially expand the use of ketamine for the treatment of high-risk clinical populations, systematic evaluation of the treatment’s effect—both single and more protracted dosing—on cognitive functioning should be undertaken. A thorough understanding of the relationship between ketamine and cognition will begin with longitudinal examination in a large, representative sample (within or across populations such as MDD and PTSD to facilitate examination of potentially meaningful differences between them) with consideration of other relevant clinical variables including stress exposure. In light of the potential impact of cognition on functional outcomes in both MDD and PTSD, inclusion of cognitive batteries into clinical trials involving ketamine should be considered. Further, it is essential that researchers consider the impact of research design and methodology on their ability to answer questions concerning ketamine’s effect on cognition.
